# Diabetic Ketoacidosis Revealing a Severe Hypertriglyceridemia and Acute Pancreatitis in Type 1 Diabetes Mellitus

**DOI:** 10.1155/2019/8974619

**Published:** 2019-01-06

**Authors:** Fatima Zahra Zaher, Imane Boubagura, Sana Rafi, Ghizlane Elmghari, Nawal Elansari

**Affiliations:** Department of Endocrinology, Diabetes, Metabolic Diseases and Nutrition, Mohammed VI University Hospital, Marrakech, Morocco

## Abstract

Diabetic ketoacidosis (DKA) is a life-threatening acute metabolic complication occurring in patients with diabetes, especially in patients with type 1 diabetes (T1D), due to an insulin deficiency. Moderate hypertriglyceridemia is commonly observed in DKA but severe hypertriglyceridemia with a triglyceride level exceeding 10g/L is very rarely reported. We report a case of a 14-year-old boy who had type 1 diabetes for 4 years treated with insulin therapy, also having adrenal insufficiency treated with hydrocortisone who presented with ketoacidosis and excruciating abdominal pain. Investigations revealed hypertriglyceridemia at 64g/L, lipasemia at 1000 U/L, and stage E pancreatitis on abdominal CT. The patient was treated with intravenous insulin, rehydration, and fenofibrate with good clinical and biological evolution. Severe hypertriglyceridemia causing pancreatitis in type 1 diabetes mellitus is a rare but very serious complication of DKA in children.

## 1. Introduction

Diabetes ketoacidosis (DKA) is the association of hyperglycemia with serum glucose ≥200 mg/dL or 11 mmol/L, the presence of ketonemia or ketonuria and acidosis with serum bicarbonate <15 mEq/L, or venous pH < 7.3 [1]. DKA represents a common complication of T1D due to an absolute insulin deficiency; it can be the mode of revelation of type 1 diabetes in 25% of cases [2], as it can complicate the evolution of a known T1D. Severe hypertriglyceridemia is an uncommon T1D complication which is also due to insulin deficiency and which can trigger acute pancreatitis. The triad of DKA, severe hypertriglyceridemia, and acute pancreatitis have been rarely described in children, especially in those with new onset T1D. We report only the tenth observation of this triad in a child known to be diabetic for 4 years with poor glycemic control.

## 2. Case Report

**Figure 1:**
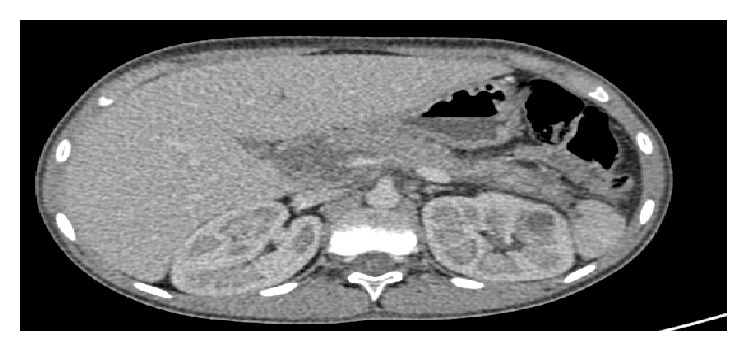
Abdominal CT showing a stage E pancreatitis.

**Figure 2:**
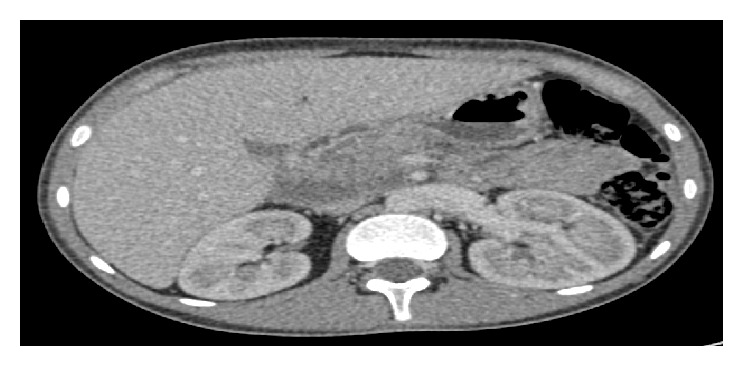
Abdominal CT showing a stage E pancreatitis.

**Figure 3:**
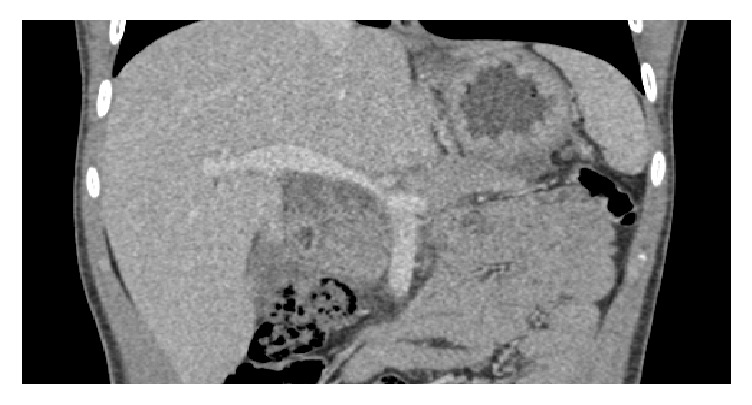
Abdominal CT showing a stage E pancreatitis.

We report a case of a 14-year-old boy with type 1 diabetes from the age of 9 years treated with insulin 4 injections per day and followed up one year ago for adrenal insufficiency treated with hydrocortisone 25mg per day. His maternal uncle had a type 2 diabetes and his sister has been diagnosed with corticoid-induced diabetes; no family history of dislipidemia was reported. This child presented to the emergency room with severe abdominal pain and vomiting and he was found to have DKA, with an elevated lipase of 1000 U/L, and his abdominal ultrasound was suggestive of acute pancreatitis associated with moderate peritoneal effusion. His abdominal computed tomography (CT) showed pancreatitis grade E with antropyloric parietal thickening extended to the 2nd part of the duodenum associated with peritoneal effusion and a moderate hepatomegaly; the imaging did not show any other anomaly causing acute pancreatitis (Figures 1, 2 and 3). His plasma triglyceride (TG) level at admission was very high 64 g/L, and C- reactive protein was elevated at 358 mg/L, and other biochemical values were at the normal range. Fluid resuscitation and insulin therapy have been started and, on arrival to the pediatric intensive care unit, he was treated with the continuous insulin infusion, fenofibrate 160mg per day, antibiotic (third-generation cephalosporins 2g per day), and hydrocortisone 50mg four times per day intravenously. The indication of plasmapheresis was not retained in the light of the reduction in triglyceride levels.

He was transferred to our department one week after stabilization. History revealed steatorrhea 15 days before admission and a glycemic imbalance with hemoglobin A1c level of 11.5% (2 months before his admission). Our patient had a Glasgow score of 15/15, pulse rate 80/min, respiratory rate 16/min, and blood pressure 90/61mmHg. Anthropometric measurements showed weight of 44 kg, height 155 cm, body mass index 18.3 kg/m2, and a capillary glycemia of 3.38 g/L with presence of urine ketone; there was no eruptive or tuberous xanthoma or xanthelasma at the skin.

The therapeutic management was continued by correction of diabetic ketosis, fenofibrates, and the use of unsaturated oils: omega 3.6.9. The evolution was favorable: clinical improvement marked by disappearance of pain and a biological improvement (Table 1).

At the follow-up, the monitoring was favorable, the level of triglycerides gradually decreases until normalization, and the last rate is 1.56 g/L.

## 3. Discussion

Diabetic ketoacidosis (DKA) is associated with an increase in triglycerides (TG) level, which is observed in approximately 30-50% of cases [3]; however severe hypertriglyceridemia (HTG), defined by a triglyceride level above 2000 mg/dL [4], is a rare complication of ketoacidosis which increases the risk of acute pancreatitis.

In many case reports, severe HTG is observed during DKA associated with newly diagnosed T1D [5, 6] or attributed to poor diabetes control [7] with severe insulin deficiency. In our case, the fact that his HbA1c was 11.5% signifies insulin deficiency for months, even if he did not report discontinuing his insulin therapy.

The mechanism of hypertriglyceridemia in DKA involves insulin deficiency which activate lipolysis in adipose tissue and increase the release of free fatty acid, which accelerate formation of very low-density lipoprotein by the liver [5]. Insulin deficiency is also associated with decreased activity of lipoprotein lipase (LPL), the enzyme responsible for TG metabolism, which reduces the clearance of VLDL and chylomicrons from the plasma, causing hypertriglyceridemia [8, 9].

However, in severe hypertriglyceridemia, the pathogenesis cannot be only explained by insulin deficiency, but may be aggravated by a coexisting genetic predisposition to hypertriglyceridemia, especially mutations in the gene coding for LPL which is located in chromosome 8 [10]. In the literature, more than 100 mutations of that gene with autosomal recessive inheritance were reported [11]. Young patients with homozygous mutation present with severe hypertriglyceridemia with abdominal pain, recurrent episodes of acute pancreatitis, and xanthomas [12] and those symptoms occur during the first year of life in approximately 25% of cases. However, in patients with a heterozygous mutation with normal health condition otherwise, blood lipid level is slightly increased or normal and disease symptoms occur during periods of insulin deficiency, stressful situation, or pregnancy [9, 11, 13].

Although severe hypertriglyceridemia is a rare condition, it may have devastating consequences such as lipidemia retinalis or acute pancreatitis [7]. Acute pancreatitis secondary to hypertriglyceridemia was reported in only 4% of DKA episodes in adults [14], and only nine cases are reported in children [5]. A triglyceride level more than 10g/L is associated with a risk of 5% for the development of acute pancreatitis; this risk is increased to 10%–20% when the triglyceride level is more than 20g/L [14]; DKA itself can cause pancreatic injury with false elevation of amylase and lipase. In our patient, the initial TG level was 64g/L. The mechanisms of this acute pancreatitis are composed of intrapancreatic release of free fatty acids resulting in cell injury, edema, and ischemia, as well as pancreatic ischemia caused directly by capillary hyperviscosity due to hyperchylomicronemia [15].

Management of DKA with HTG includes continuous insulin administration and intravenous fluid and according to diabetic ketoacidosis guidelines because the major mechanism of hypertriglyceridemia is insulin deficiency. However, some patients may require plasmapheresis, especially those with organ failure, which is the case of the patient reported by Lutfi who had extremely high TG level (163 g/L) with renal insufficiency, who failed with conservative treatment, but was successfully treated by plasmapheresis [16]. Heparin was also reported as a treatment for hypertriglyceridemia in adult with DKA, associated with fluid and insulin administration [17]. The use of lipid-lowering agents, such as fibrates, can be useful in most conditions accompanied by HTG but not beneficial in patients with lipoprotein lipase deficiency [18]. Omega oils are essential fatty acid naturally present in fish, algae, and other seafood; their use as a TG-lowering drug in the management of severe HTG is recommended by both the National Lipid Association [19] and the American Heart Association [20]. The EVOLVE study found a statistically significant reduction in serum TG in patients with severe HTG treated with omega 3 oil 2 g daily in comparison with those treated with olive oil 2 g daily, with a greater treatment effect in patients with very high baseline TG concentrations [21]. The proposed mechanisms to explain these finding include decreased VLDL synthesis by altering transcription factors such as sterol regulatory element-binding proteins involved in triglyceride synthesis and increased triglyceride clearance from the serum by increased lipoprotein lipase activity [22]. In our case, TG level was reduced from 64g/l to 3.37 g/L in 5 days, under insulin, fluid administration, fibrates, and omega oils.

The weakness of our case was that we could not perform appropriate genetic testing in our patient as well as his first-degree relatives. The genetic testing would have had an impact on the management of our patient since the majority of TG-lowering drugs, other than omega oils, decrease the plasma TG levels by the stimulation LPL activity, which makes them less effective in the case of a severely dysfunctional LPL protein. Surendran et al. have shown that a molecular diagnosis is effective in patients with severe HTG, and, by diagnosing the underlying genetic defect, different therapeutic strategies can be proposed [23]. For example, in the case of a genetic defect in GPIHBP1, where LPL itself is unaffected, Surendran et al. successfully used heparin infusion to facilitate activating LPL in the absence of GPIHBP1. On the other hand, in the case of patients with genetic LPL gene deficiency, LPL gene therapy has been used successfully. However, in the case of APOC2 or LMF1 deficiency, no clear treatment options are currently available, beyond strict dietary restrictions [23].

## 4. Conclusion

In patients with diabetic ketoacidosis and acute pancreatitis, clinicians should be aware of the possibility of associated hypertriglyceridemia caused by the severe insulin deficiency, recognition of this triad has important implications in the management of the patient as insulin requirements, and recovery period can be changed.

## Figures and Tables

**Table 1:** Evolution of biological parameters.

**DAYS**	**TG (g/l)**	**LIPASE (U/l)**	**CRP (mg/l)**
*1st day*	64	1000	358
*2nd day*	20	410	350
*3rd day*	12	285	169
*4th day*	4.31	-	76
*5th day*	3.37	159	20
*6th day*	4.20	-	5.38
*7th day*	3.32	65	5.25
